# Consultation-liaison psychiatry in Japan: a nationwide retrospective observational study

**DOI:** 10.1186/s12888-021-03241-y

**Published:** 2021-05-05

**Authors:** Daisuke Shinjo, Hisateru Tachimori, Keiko Maruyama-Sakurai, Kenji Fujimori, Norihiko Inoue, Kiyohide Fushimi

**Affiliations:** 1 Department of Information Technology and Management, The National Center of Child Health and Development, Tokyo, Japan; 2 Department of Health Policy and Informatics, Tokyo Medical and Dental University Graduate School, 1-5-45 Yushima, Bunkyo-ku, Tokyo, 1138519 Japan; 3 Department of Mental Health Policy and Evaluation, National Institute of Mental Health, The National Center of Neurology and Psychiatry, Tokyo, Japan; 4 Institute for Global Health Policy Research, The National Center for Global Health and Medicine, Tokyo, Japan; 5 Department of Health Administration and Policy, Tohoku University, Sendai, Japan

**Keywords:** Consultation-liaison psychiatry, Geographic disparity, Administrative database, DPC, Japan

## Abstract

**Background:**

Consultation-liaison psychiatry (CLP)—professional psychiatric care provided to coordinate with surgical or medical treatment of inpatients with psychiatric disorders—was included in universal health coverage in Japan in 2012. Despite evidence of benefits of CLP, basic data and geographic distribution information regarding CLP at the national level remain unclear. This study aimed to 1) identify the geographic disparity of CLP in Japan and 2) investigate the association between number of consultations per CLP patient and region.

**Methods:**

We retrospectively analyzed anonymized data retrieved from the Japanese administrative inpatient database regarding inpatients who were provided CLP between April 2012 and March 2017. Demographic characteristics were summarized and geographic disparity by prefecture was visualized for fiscal years 2012 and 2016; we also summarized the data according to region. Multivariate linear regression analysis was used to investigate association between the number of consultations per CLP patient and region after adjusting for covariates.

**Results:**

Data from a total of 46,171 patients who received 138,866 CLP services were included. Results revealed more patients aged 75–84 years received CLPs than any other age group (29.7%) and the overall male/female ratio was 53:47 in 2016. In 2012 and 2016, 24.2 and 30.7% of CLP patients, respectively, were transferred to other hospitals; 9.7 and 8.8%, respectively, discharged due to the death. CLP services were provided in 14 prefectures in 2012 and 33 by 2016; 14 prefectures had no available CLP services. After adjusting for covariates, Tohoku (β = − 0.220, *p* < 0.034), Chugoku (β = − 0.160, *p* < 0.026), and Shikoku (β = − 0.555, *p* < 0.001) had a significant negative correlation with the number of consultations per CLP patient compared with Hokkaido region (an adjusted R square (R2) = 0.274).

**Conclusions:**

Our study clarified the characteristics of patients in Japan who received CLPs and the geographic disparity in CLP services. Although 5 years had passed since CLP was introduced, the results imply wide availability of CLP nationally. The analysis data provided may inform future policies to improve CLP services.

**Supplementary Information:**

The online version contains supplementary material available at 10.1186/s12888-021-03241-y.

## Background

Across OECD (Organization for Economic Cooperation and Development) member countries, on average, the share of the population aged 65 and over is projected to continue increasing in the coming decades, which will likely lead to greater demand for labor-intensive long-term healthcare [1]. As societies are progressively aging and healthcare technology is advancing, the number of medical or surgical patients with psychiatric comorbidities is increasing. This increase is due, in part, to the higher risk of mental disorders in elderly populations than other populations [2]. Additionally, hospital inpatients with any psychiatric comorbidity are more likely to utilize health care resources (i.e. longer hospital stays) than those with medical conditions only [3–6]. Indeed, psychological comorbidities commonly exacerbate physical illness-associated symptoms, contributing to increased healthcare utilization and costs. Psychiatric comorbidity is even associated with increasing excess mortality [7, 8] and the number of patient rehospitalizations [9].

Consultation-liaison psychiatry (CLP) is the discipline of providing professional psychiatric care to inpatients who are primarily admitted for medical or surgical (somatic) reasons in whom comorbid psychiatric symptoms are revealed after hospital admission. From the reports by Lipowski [10–12], the scope, organization, and strategic aspects of CLP in general hospitals/wards have developed worldwide. Previous studies show that CLP reduces medical complications, length of stay, and number of hospitalizations via early referral for psychiatric consultations, treatment of psychiatric presentation on medical and surgical units, and facilitating access to appropriate psychiatric treatment post-discharge [13–17].

In terms of implementing CLP services and in the application of CLP guidelines, there are several differences among countries [18]. In Japan, the concept of CLP was introduced over 50 years ago, but was in preliminary stages of development for many years [18]; CLP was included in the health insurance standards in 2012, thus it was finally covered by universal health coverage. This inclusion was due to the growing needs for psychiatric care to patients with psychiatric disorders such as delirium in acute-care hospitals. Also, this inclusion was partly based on both the request from associations of healthcare providers and political motivation for supporting interprofessional team collaboration for improving quality of care and patients’ quality of life.

In short, CLP care is to be provided by a multidisciplinary team (i.e. psychiatrists, nurses, pharmacists, and other professions) to improve quality of care and treatment in medical and surgical patients who may benefit from coordinated, multi-discipline care. The CLP team organized treatment plan based on the CLP guidance published by the Japanese Society of General Hospital Psychiatry [19]. On CLP, psychiatrists and psychiatric-trained staff may get involved from the beginning and contribute to prevention, earlier detection, and intervention for the psychological distress and psychiatric symptoms [20]. CLP may also provide education to the staff team, provide direct care to the patients’ family, and adjust psychiatric care after discharge. The details of CLP services in Japan were described elsewhere [18]. CLP would enable us to find such patients and provide psychiatric care in their early stage, which contribute to reducing their psychiatric symptoms and assisting early discharge.

Previous studies have reported several aspects of CLP services in Japanese settings, such as in cancer cohorts, psychiatric diagnoses, and treating depression [17, 21, 22]. However, neither basic data nor geographic disparity pertaining to the provision of CLP services in the national level remains unclear. It is essential to capture clinical features of patients who received CLP services and CLP distribution for achieving efficient CLP care under the limited healthcare resources available to the rapidly aging society. Use of Nationwide Database is one of the attractive approaches to address this issue.

The aim of this study was to use information from the Japanese Administrative Database to describe the clinical features of current CLP settings and to reveal the geographic distribution of CLP services in Japan. This study was also designed to investigate the association between the number of consultations per CLP patient and region after adjusting for covariates.

## Methods

### Data source

This was a retrospective, observational study that used data from the Japanese Administrative Database; the Diagnosis Procedure Combination (DPC) per-diem payment system (DPC/PDPS) (details of the DPC/PDPS have been described elsewhere) [23, 24]. Briefly, the DPC/PDPS is a case-mix patient classification system that is linked to payments at acute-care and mixed-care hospitals in Japan. By 2016, the DPC/PDPS-based hospital reimbursement system had been adopted by more than 1600 hospitals, which accounted for more than half of the total 894,000 hospital beds nationwide. The DPC (Administrative) database, consists of routinely collected electronic data, is different from registry database where patients who meet criteria are registered according to the study purpose by the healthcare professional.

Anonymous clinical and administrative claims data were collected annually for patients from the participating hospitals. Clinical data consists of baseline patient information, diagnosis (based on ICD-10), and detailed medical information such as all major or minor procedures, medication, and device use. The database also includes the purpose of admission, discharge destination, and outcome at the time of hospital discharge. Hospital information is also collected under the DPC/PDPS. We obtained population data according to prefectures from a national survey called Population Estimates [25]. Each region consists of several prefectures. The database did not include detailed clinical data regarding psychiatry.

This study was approved by the Institutional Review Board at the Tokyo Medical and Dental University and the National Center for Child Health and Development. The board waived off the requirement for patient informed consent because of the anonymous nature of the data. Data used in this study is not publicly available (for further information, see Declarations “Ethics approval and consent to participate”).

### Participants and variables

We identified patients who had received CLP (Japanese code: A230–4) between April 1, 2012, and March 31, 2017, from the DPC database. We excluded 224 patients with 397 CLP services from one hospital due to not having complete hospital information.

Data pertaining to individual-level characteristics were extracted. Individual variables included age, sex, admission status (planned, unplanned, or urgent), discharge settings, discharge outcome, and disease classification according to ICD-10. Age was categorized into six groups: 0–29, 30–49, 50–64, 65–74, 75–84, and 85+. Disease classification was categorized according to ICD-10 Chapter numbers from “I “(Certain infectious and parasitic diseases) to “XXII’ (Codes for special purposes). Data regarding in-hospital psychotherapy were also obtained from the database. In-hospital psychiatry was recorded in the administrative database a maximum of three times per week due to the limitations of the payment system. In this study, in-hospital psychotherapy is defined as a Japanese medical service fee code I001, a psychological therapy for inpatients with psychiatric disorders, provided by the psychiatrists according to the assessment of the patients. Each psychotherapy session must last for more than 30 minutes as “I100 in-hospital psychotherapy,” and claimed for a maximum of 3 times per week (depending on a patient’s condition).

### Statistical analysis

Continuous variables were summarized with the use of descriptive statistics (mean ± standard deviation for values with normal distribution, and the median with interquartile range (IQR) for values with non-normal distribution), and categorical variables were summarized as frequencies and proportions. Chi-square test of independence with Cramer’s V strength was used to investigate if there is a significant relationship between regions and other variables. An analysis of variance was used to determine whether there are any statistically significant differences between the means of regions. Post hoc analyses were performed using the Scheffé method or Fisher’s Exact test. A forced entry multivariate linear regression analysis was conducted to determine if region was independently correlated with the number of consultations per CLP patient after adjusting for other variables. The variance inflation factor (VIF) was used for checking the degree of multicollinearity. All statistical analyses were performed using R statistical software, version 3.3.2 (R Foundation for Statistical Computing, Vienna, Austria), and data visualization were performed by Tableau Software version 2018.3 (Tableau software, Seattle, United States of America).

## Results

### Patient and hospital characteristics

A total of 46,171 patients who were provided CLP qualified based on the inclusion criteria. Table 1 shows the basic characteristics of those patients and hospitals. The age group who used the services the most included those patients aged 75–84 years (29.7% in 2016). About half of all patients were female (47.2% in 2016), and the proportion of patients whose CLP-related medical care was unplanned or urgent was 67.8% in 2016. Also in that year, 109 hospitals in Japan provided CLPs. The number of provided CLP services, patients who received CLP, and hospitals providing CLP increased almost three- to four-fold in between 2012, when CLP was introduced, and 2016 (428, 406, and 363%, respectively). The frequency of CLP per patient did not change significantly during the study period. The coefficient of variation of the number of consultations per CLP patient remained relatively constant during our study period.

**Table 1:** Characteristics of patients and hospitals cohorts in the study (Patients *N* = 46,170)

Variables	Fiscal year					Proportion (2012)	Proportion (2016)	Rate of increase (2016 vs. 2012)
	2012	2013	2014	2015	2016			
Patients	3989	5352	8358	11,408	17,064			428%
Consultation-Liaison Psychiatry								
Overall	11,800	17,807	26,283	35,084	47,912			406%
Per patient (mean, SD)	2.96 (2.73)	3.33 (3.64)	3.14 (3.14)	3.08 (3.15)	2.81 (2.75)			–
Per patient (median, interquartile range)	2 [1–4]	2 [1–4]	2 [1–4]	2 [1–4]	2 [1–3]			–
Per patient (range)	1–29	1–67	1–40	1–48	1–80			
Coefficient of variation	0.92	1.10	1.00	1.03	0.98			–
Hospitals	30	34	42	51	109			363%
Age group								
0–29	164	231	327	479	662	4.1%	3.9%	
30–49	542	796	1193	1299	2019	13.6%	11.8%	
50–64	721	1013	1431	1795	2556	18.1%	15.0%	
65–74	778	1127	1746	2536	3527	19.5%	20.7%	
75–84	1168	1391	2386	3279	5061	29.3%	29.7%	
85+	616	794	1275	2020	3239	15.4%	19.0%	
Sex								
Male	2026	2651	4290	6023	9015	50.8%	52.8%	
Female	1963	2701	4068	5385	8049	49.2%	47.2%	
Admission setting								
Planned	1451	1887	3023	4051	5497	36.4%	32.2%	
Unplanned or Urgent	2538	3465	5335	7357	11,567	63.6%	67.8%	
Discharge setting								
Outpatients follow up	2413	3209	4730	6255	9100	60.5%	53.3%	
Transfer to other hospitals	966	1311	2231	3334	5238	24.2%	30.7%	
Transfer to other welfare facilities	139	173	486	611	876	3.5%	5.1%	
Others	471	659	911	1208	1850	11.8%	10.8%	
Discharge outcome								
Treated	3177	4331	6709	9370	14,025	79.6%	82.2%	
Unchanged or worsened	358	333	671	789	1124	9.0%	6.6%	
Death	385	539	783	985	1505	9.7%	8.8%	
Other	69	149	195	264	410	1.7%	2.4%	
Length of stay								
2–17 days	989	1189	2294	3179	4754	24.8%	27.9%	
18–30 days	938	1269	1941	2724	4330	23.5%	25.4%	
31–54 days	1038	1315	1952	2798	4211	26.0%	24.7%	
55 days and over	1024	1579	2171	2707	3769	25.7%	22.1%	

Consultation-Liaison Psychiatry (CLP) services were recorded once a week regardless of multiple CLPs being providing in a week

The characteristics of patients’ discharge settings and outcomes as of discharge are also shown in Table 1. More than half of the patients were discharged and followed-up by outpatient services (53.3% in 2016), while about nearly one-third of the patients were transferred to other hospitals (30.7% in 2016) and 5.1% of were transferred to welfare facilities in 2016. Overall, the in-hospital mortality ratio for the patients who received CLP services was about 9% (8.8% in 2016).

Characteristics of disease classification by “most resource-consuming diagnosis,” of study cohorts are summarized in Table 2. The highest proportion of disease classification was “neoplasm” (22.9% in 2016), followed by “disease of circulatory system” (16.7% in 2016), “injury, poisoning and certain other consequences of external causes” (13.7% in 2016) and “other” classifications.

**Table 2:** Disease classification of the study cohorts

Disease classification	Fiscal year					Proportion of patients (n) in 2012	Proportion of patients (n) in 2016
	2012	2013	2014	2015	2016		
1. Certain infectious and parasitic diseases	146	187	345	377	580	3.7%	3.4%
2. Neoplasms	1083	1333	2036	2795	3906	27.1%	22.9%
3. Diseases of the blood and blood-forming organs and certain disorders involving the immune mechanism	70	97	131	166	226	1.8%	1.3%
4. Endocrine, nutritional, and metabolic diseases	164	232	320	385	596	4.1%	3.5%
5. Mental and behavioral disorders	65	107	172	181	328	1.6%	1.9%
6. Diseases of the nervous system	141	189	325	393	609	3.5%	3.6%
7. Diseases of the eye and adnexa	25	26	41	38	71	0.6%	0.4%
8. Diseases of the ear and mastoid process	7	9	36	40	39	0.2%	0.2%
9. Diseases of the circulatory system	614	788	1264	1870	2851	15.4%	16.7%
10. Diseases of the respiratory system	365	493	723	912	1542	9.2%	9.0%
11. Diseases of the digestive system	352	499	782	1046	1666	8.8%	9.8%
12. Diseases of the skin and subcutaneous tissue	59	98	173	194	261	1.5%	1.5%
13. Diseases of the musculoskeletal system and connective tissue	171	302	517	727	1085	4.3%	6.4%
14. Diseases of the genitourinary system	168	241	371	552	711	4.2%	4.2%
15. Pregnancy, childbirth, and the puerperium	52	67	120	131	213	1.3%	1.2%
17. Congenital malformations, deformations, and chromosomal abnormalities	10	13	21	31	40	0.3%	0.2%
18. Symptoms, signs, and abnormal clinical and laboratory findings, not elsewhere classified	1	2	3	8	6	0.0%	0.0%
19. Injury, poisoning, and certain other consequences of external causes	496	669	978	1562	2333	12.4%	13.7%
21. Factors influencing health status and contact with health services	0	0	0	0	1	0.0%	0.0%

The provision of in-hospital psychotherapy in the study cohort was summarized in Appendix. About three-tenth of the patients did not have in-hospital psychotherapy (31.9% in 2016), while about one-fifth of the patients had just one in-hospital psychotherapy (19.5% in 2016). Almost half of the patients had several in-hospital psychotherapy sessions, and 13.9% of the patients had more than six psychotherapy sessions in 2016.

**Fig. 1:**
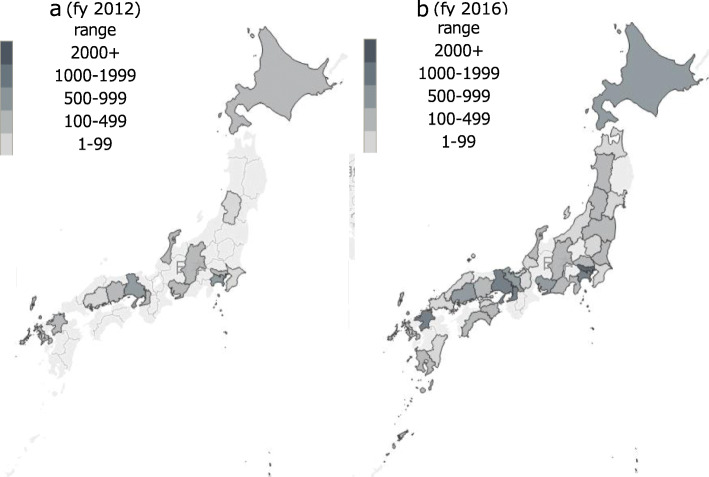
Geographic distribution of Consultation-liaison psychiatry in 2012 and 2016. The figure was drawn by using the Tableau software version 2018.3 with its built-in map of Japan

The geographic distribution of CLP services in 2012 and 2016 are shown in Fig. 1. Among the 47 prefectures in Japan, CLPs were provided in 14 prefectures (29.8%) at 30 hospitals in 2012. As of 2016, CLPs were provided in 33 prefectures (70.2%) at 109 hospitals; 14 prefectures have never offered CLPs. Table 3 also shows descriptive statistics of CLPs by region. There was no hospital providing CLP services in the Shikoku area until 2014 (data not shown), while more than one-third of the CLP services provided in 2016 were in the Kanto area. The provided CLP services per population ratio showed wide variation even in the regional area level (range: 0.19–0.54 CLP services per thousand population).

**Table 3:** Cross tabulation for number of patients, provided CLP services, hospitals, and population data by region

	Number of patients		Number of CLP performed		Number of hospitals providing CLP		Number of DPC hospitals	Regional data						CLP services per population
	Fiscal year		Fiscal year		Fiscal year		Fiscal year	Number of prefectures			populations (2016) *			
**Region**	**2012**	**2016**	**2012**	**2016**	**2012**	**2016**	**2016**	**overall**	**CLP in 2012**	**CLP in 2016**	**overall**	**65+**	**aging ratio**	**2016**
Hokkaido	202	939	673	2772	1	4	89	1	1	1	5352	1602	29.9%	0.52
Tohoku	35	641	115	1879	1	10	109	6	1	5	8915	2674	30.0%	0.21
Kanto	1008	6138	3016	18,309	11	35	427	7	3	6	43,132	10,748	24.9%	0.42
Chubu	743	1512	2209	4175	4	19	274	9	3	6	21,415	5922	27.7%	0.19
Kinki	1100	4561	3185	12,038	8	22	325	7	2	4	22,489	6203	27.6%	0.54
Chugoku	268	774	900	2007	3	6	116	5	2	4	7406	2218	29.9%	0.27
Shikoku		411		925		4	64	4	0	3	3818	1209	31.7%	0.24
Kyushu-Okinawa	633	2088	1702	5807	2	9	262	8	2	4	14,405	4014	27.9%	0.40
Total	3989	17,064	11,800	47,912	30	109	1666	47	14	33	126,933	34,591	27.3%	0.38

*CLP* Consultation-Liaison Psychiatry, *DPC* Diagnosis Procedure Combination; ^*^ unit: thousands

The result of the test of independence and ANOVA (Fiscal year 2016) is shown in Tables 4 and 5 (Post hoc analysis was presented in Additional file 1). The result revealed there is an association between regions and other variables; age group (*p* < 0.001, Cramer’s V = 0.097), sex (*p* = 0.009, Cramer’s V = 0.032), admission setting (p < 0.001, Cramer’s V = 0.163), discharge setting (*p* < 0.001, Cramer’s V = 0.127), discharge outcome (*p* < 0.001, Cramer’s V = 0.077), disease classification (*p* < 0.001, Cramer’s V = 0.078), length of stay (*p* < 0.001, Cramer’s V = 0.044) and number of CLP services per patient (*p* < 0.001). Number of patients aged over 75 was the highest in Tohoku (63.1%) and lowest in Kyusyu-Okinawa (38.9%). The proportion of female patients was slightly lower than that of male patients. In most case, the admission setting was unplanned (52.9 to 84.4%). Shikoku (56.4%) and Kyusyu-Okinawa (55.0%) were characterized by a higher proportion of patients who transferred to other hospitals. The discharge outcome was mostly “treated” (78.5 to 90.8%), followed by “death” (5.6 to 11.0%). CLP per patient was highest in Kanto (mean = 2.98).

**Table 4:** Characteristics of patients by region (2016)

Variables	Hokkaido	Tohoku	Kanto	Chubu	Kinki	Chugoku	Shikoku	Kyushu-Okinawa	*p* value	Cramer’s V
N	939	641	6138	1512	4561	774	411	2088		
Age group (%)									< 0.001	0.097
0–29	42 (4.5)	12 (1.9)	291 (4.7)	41 (2.7)	128 (2.8)	16 (2.1)	5 (1.2)	127 (6.1)		
30–49	116 (12.4)	45 (7.0)	873 (14.2)	149 (9.9)	417 (9.1)	76 (9.8)	37 (9.0)	306 (14.7)		
50–64	159 (16.9)	77 (12.0)	1070 (17.4)	191 (12.6)	522 (11.4)	98 (12.7)	61 (14.8)	378 (18.1)		
65–74	228 (24.3)	103 (16.1)	1275 (20.8)	274 (18.1)	942 (20.7)	153 (19.8)	87 (21.2)	465 (22.3)		
75–84	283 (30.1)	212 (33.1)	1724 (28.1)	480 (31.7)	1469 (32.2)	260 (33.6)	128 (31.1)	505 (24.2)		
85+	111 (11.8)	192 (30.0)	905 (14.7)	377 (24.9)	1083 (23.7)	171 (22.1)	93 (22.6)	307 (14.7)		
Sex (%)									0.009	0.032
Male	506 (53.9)	329 (51.3)	3346 (54.5)	784 (51.9)	2327 (51.0)	394 (50.9)	234 (56.9)	1095 (52.4)		
Female	433 (46.1)	312 (48.7)	2792 (45.5)	728 (48.1)	2234 (49.0)	380 (49.1)	177 (43.1)	993 (47.6)		
Admission setting (%)									< 0.001	0.163
Planned	442 (47.1)	100 (15.6)	2157 (35.1)	336 (22.2)	1445 (31.7)	253 (32.7)	66 (16.1)	698 (33.4)		
Unplanned or Urgent	497 (52.9)	541 (84.4)	3981 (64.9)	1176 (77.8)	3116 (68.3)	521 (67.3)	345 (83.9)	1390 (66.6)		
Discharge setting (%)									< 0.001	0.127
Outpatients follow up	600 (63.9)	302 (47.1)	3648 (59.4)	746 (49.3)	2390 (52.4)	363 (46.9)	133 (32.4)	918 (44.0)		
Transfer to other hospitals	248 (26.4)	203 (31.7)	1586 (25.8)	449 (29.7)	1324 (29.0)	278 (35.9)	232 (56.4)	918 (44.0)		
Transfer to other welfare facilities	15 (1.6)	61 (9.5)	247 (4.0)	137 (9.1)	277 (6.1)	33 (4.3)	22 (5.4)	84 (4.0)		
Others	76 (8.1)	75 (11.7)	657 (10.7)	180 (11.9)	570 (12.5)	100 (12.9)	24 (5.8)	168 (8.0)		
Discharge outcome									< 0.001	0.077
Treated	812 (86.5)	539 (84.1)	4817 (78.5)	1242 (82.1)	3824 (83.8)	639 (82.6)	373 (90.8)	1779 (85.2)		
Unchanged or worsened	35 (3.7)	34 (5.3)	555 (9.0)	95 (6.3)	239 (5.2)	46 (5.9)	15 (3.6)	105 (5.0)		
Death	69 (7.3)	68 (10.6)	568 (9.3)	153 (10.1)	399 (8.7)	85 (11.0)	23 (5.6)	140 (6.7)		
Other	23 (2.4)	0 (0.0)	198 (3.2)	22 (1.5)	99 (2.2)	4 (0.5)	0 (0.0)	64 (3.1)		
Length of stay									< 0.001	0.044
2–17 days	297 (31.6)	134 (20.9)	1650 (26.9)	358 (23.7)	1380 (30.3)	205 (26.5)	119 (29.0)	611 (29.3)		
18–30 days	233 (24.8)	150 (23.4)	1452 (23.7)	397 (26.3)	1217 (26.7)	220 (25.8)	125 (30.4)	556 (26.6)		
31–54 days	189 (20.1)	173 (27.0)	1539 (25.1)	409 (27.1)	1093 (24.0)	221 (28.6)	87 (21.1)	500 (23.9)		
55 days and over	220 (23.4)	184 (28.7)	1497 (24.4)	348 (23.0)	871 (19.1)	148 (19.1)	80 (19.5)	421 (20.2)		
Consultation-Liaison Psychiatry per patient (mean (sd))	2.95 (2.83)	2.93 (2.63)	2.98 (2.89)	2.76 (2.46)	2.64 (2.54)	2.59 (2.25)	2.25 (1.90)	2.78 (3.22)	< 0.001

**Table 5:** Disease classification of patients by region (2016)

Variables	Hokkaido	Tohoku	Kanto	Chubu	Kinki	Chugoku	Shikoku	Kyushu-Okinawa	*p* value	Cramer’s V
N	939	641	6138	1512	4561	774	411	2088		
Disease classification									< 0.001	0.078
1. Certain infectious and parasitic diseases	33 (3.5)	18 (2.8)	230 (3.7)	46 (3.0)	157 (3.4)	16 (2.1)	10 (2.4)	70 (3.4)		
2. Neoplasms	240 (25.6)	104 (16.2)	1393 (22.7)	302 (20.0)	1060 (23.2)	234 (30.2)	59 (4.4)	514 (24.6)		
3. Diseases of the blood and blood-forming organs and certain disorders involving the immune mechanism	19 (2.0)	9 (1.4)	84 (1.4)	15 (1.0)	68 (1.5)	7 (0.9)	4 (1.0)	20 (1.0)		
4. Endocrine, nutritional, and metabolic diseases	30 (3.2)	18 (2.8)	216 (3.5)	59 (3.9)	160 (3.5)	17 (2.2)	12 (2.9)	84 (4.0)		
5. Mental and behavioral disorders	13 (1.4)	11 (1.7)	107 (1.7)	35 (2.3)	72 (1.6)	23 (3.0)	8 (1.9)	59 (2.8)		
6. Diseases of the nervous system	44 (4.7)	13 (2.0)	238 (3.9)	52 (3.4)	133 (2.9)	34 (4.4)	12 (2.9)	83 (4.0)		
7. Diseases of the eye and adnexa	10 (1.1)	3 (0.5)	20 (0.3)	14 (0.9)	8 (0.2)	3 (0.4)	0 (0.0)	13 (0.6)		
8. Diseases of the ear and mastoid process	2 (0.2)	3 (0.5)	26 (0.4)	3 (0.2)	2 (0.0)	0 (0.0)	0 (0.0)	3 (0.1)		
9. Diseases of the circulatory system	191 (20.3)	125 (19.5)	1219 (19.9)	230 (15.2)	710 (15.6)	119 (15.4)	67 (16.3)	190 (9.1)		
10. Diseases of the respiratory system	58 (6.2)	72 (11.2)	502 (8.2)	185 (12.2)	426 (9.3)	59 (7.6)	78 (19.0)	162 (7.8)		
11. Diseases of the digestive system	72 (7.7)	70 (10.9)	557 (9.1)	147 (9.7)	480 (10.5)	81 (10.5)	37 (9.0)	222 (0.6)		
12. Diseases of the skin and subcutaneous tissue	12 (1.3)	8 (1.2)	83 (1.4)	33 (2.2)	64 (1.4)	19 (2.5)	4 (1.0)	38 (1.8)		
13. Diseases of the musculoskeletal system and connective tissue	56 (6.0)	27 (4.2)	439 (7.2)	72 (4.8)	338 (7.4)	29 (3.7)	16 (3.9)	108 (5.2)		
14. Diseases of the genitourinary system	41 (4.4)	28 (4.4)	229 (3.7)	77 (5.1)	217 (4.8)	24 (3.1)	13 (3.2)	82 (3.9)		
15. Pregnancy, childbirth, and the puerperium	15 (1.6)	8 (1.2)	67 (1.1)	18 (1.2)	40 (0.9)	2 (0.3)	1 (0.2)	62 (3.0)		
17. Congenital malformations, deformations, and chromosomal abnormalities	3 (0.3)	1 (0.2)	22 (0.4)	2 (0.1)	4 (0.1)	4 (0.5)	2 (0.5)	2 (0.1)		
18. Symptoms, signs, and abnormal clinical and laboratory findings, not elsewhere classified	0 (0.0)	0 (0.0)	2 (0.0)	0 (0.0)	2 (0.0)	0 (0.0)	1 (0.2)	1 (0.0)		
19. Injury, poisoning, and certain other consequences of external causes	100 (10.6)	123 (19.2)	704 (11.5)	222 (14.7)	620 (13.6)	103 13.3)	87 (21.2)	374 (17.9)		
21. Factors influencing health status and contact with health services	0 (0.0)	0 (0.0)	0 (0.0)	0 (0.0)	0 (0.0)	0 (0.0)	0 (0.0)	1 (0.0)		

Table 6 shows the results of a multiple regression analysis wherein the number of consultations per CLP patient was used as the dependent variable. Multivariate linear regression analysis revealed that Shikoku (β = − 0.024, *p* < 0.001), Chugoku (β = − 0.023, *p* < 0.001), and Kyusyu-Okinawa (β = − 0.030, *p* < 0.001) had a significant negative correlation with the number of consultations per CLP patient compared with Hokkaido region, after adjusting for age group, sex, admission setting, disease classification, length of stay, and fiscal year. Adjusted R square (R2) of the model was 0.274, which indicates that this model explains 27.4% of the variance in the dependent variable. No strong collinearity was observed among the explanatory variables (VIF < 2 for each explanatory variable).

**Table 6:** Multivariate linear regression analysis results for number of consultations per CLP patient

Variable	β	*p* value	VIF
Region (reference: Hokkaido)			1.012
Tohoku	−0.220	0.034	
Kanto	−0.075	0.192	
Chubu	−0.071	0.279	
Kinki	0.009	0.882	
Chugoku	−0.160	0.026	
Shikoku	−0.555	< 0.001	
Kyusyu-Okinawa	−0.121	0.055	
Age group (reference: 85+)			1.027
0–29	0.410	< 0.001	
30–49	0.263	< 0.001	
50–64	0.256	< 0.001	
65–74	0.144	< 0.001	
75–84	0.016	< 0.001	
Sex (reference: Male)			1.030
Female	0.048	0.011	
Admission setting (reference; planed)			1.154
Unplanned	−0.021	0.044	
Disease classification (reference: 9.Diseases of the circulatory system)			1.014
1.Certain infectious and parasitic diseases	0.315	< 0.001	
2.Neoplasms	0.098	0.018	
3.Diseases of the blood and blood-forming organs and certain disorders involving the immune mechanism	0.298	0.004	
4.Endocrine, nutritional, and metabolic diseases	−0.025	0.722	
5.Mental and behavioral disorders	−1.016	< 0.001	
6.Diseases of the nervous system	0.076	0.283	
7.Diseases of the eye and adnexa	−0.127	0.498	
8.Diseases of the ear and mastoid process	−0.287	0.212	
10.Diseases of the respiratory system	0.133	0.010	
11.Diseases of the digestive system	0.088	0.079	
12.Diseases of the skin and subcutaneous tissue	0.118	0.225	
13.Diseases of the musculoskeletal system and connective tissue	0.290	< 0.001	
14.Diseases of the genitourinary system	−0.007	0.912	
15.Pregnancy, childbirth, and the puerperium	−0.295	0.012	
17.Congenital malformations, deformations, and chromosomal abnormalities	−0.121	0.622	
18.Symptoms, signs, and abnormal clinical and laboratory findings, not elsewhere classified	0.053	0.927	
19.Injury, poisoning, and certain other consequences of external causes	0.125	0.006	
21.Factors influencing health status and contact with health services	−0.392	0.880	
Fiscal year (reference; 2012)			1.010
2013	0.226	< 0.001	
2014	0.235	< 0.001	
2015	0.233	< 0.001	
2016	0.039	0.398	
Length of Stay (reference; 2–17 days)			1.015
18–30 days	0.111	< 0.001	
31–54 days	0.246	< 0.001	
55 days and over	0.587	< 0.001	
Adjusted R-squared	0.274		

*VIF* Variance Inflation Factor

## Discussion

The present retrospective study investigated the national-level CLP service in general hospitals in Japan, using a national inpatient database. The present study characterized (1) the key overview of patients who received CLP services and (2) the geographic disparity of these patients. To the best of our knowledge, this article is one of the first reports revealing CLP disparity. CLP would contribute to not only providing psychiatric care for patients admitted to medical general wards, but also promoting patients’ smooth discharge through early detection and intervention for the psychological and psychiatric symptoms. CLP also helped to adjust psychiatric care after discharge, which may lead patients to be promoted appropriate adherence and be treated within the psychiatric community-care networks that have a significant impact on the use of mental healthcare services [26]. In today’s current health care climate of cost savings, limited allocation of resources, and expectations of demonstrations of the value of services and clinical productivity, it is important to clarify the current situation regarding CLP services to understand how to make for future improvements to the healthcare system.

After the introduction of CLP services in 2012, the provision of CLP was consistently increased (Table 1), implying the recognition of the need for CLP services in medical and surgical patients. Also, almost 70% of patients we studied who received CLP services were over the age of 65 in 2016, which was much higher than in recent studies in a Canadian setting (roughly 42% from two academic tertiary care hospitals) [27] and an Italian setting (mean and SD age was 57.9 ± 19.4 from one general hospital) which were in line with other reports from Europe in terms of demographic data (about 41%) [28]. We speculated that this is due to the difference in the aging ratio of the study population, criteria for referral, priority/availability for CLP referral, and the healthcare system. Our results also showed that about 70% of CLP services were provided to patients whose admittance was unplanned or who were admitted due to an urgent condition. This is partly because such patients may not have been prepared for, or may be especially agitated about, their health problems compared with planned admission patients.

While data regarding discharge settings and outcomes were usually unavailable internationally, it was reasonable that about three out of every 10 patients who received CLP were transferred to other hospitals (Table 1), which is much higher than the 5.8–7.5% overall average of acute−/mixed-care inpatients in a Japanese setting [29]. We speculated that some of the patients with psychiatric comorbidities were transferred to psychiatric hospitals (detailed data about discharge settings were not available). It was surprising that about 9 % of patients who received CLP services were discharged as dead (Table 1), which was also much higher than the overall average of 1.7–3.3% [30]. We also speculated that some CLP services were provided for severe patients who needed psychiatric support for improving their mental condition as a part of end-of-life care. However, further studies are required to address this issue due to data unavailability.

It was reasonable that more than one-fifth of CLP services were provided to the cancer patients, considering both the number of cancer inpatients (13.4% in general hospitals) [31] and that approximately 29–43% of these patients fulfilled the diagnostic criteria for having a psychiatric disorder [32, 33] (Table 2). However, in terms of international comparisons of CLP data, it is not easy to compare in detail. For example, few data were available in the basic disease classifications of the study cohorts. Even if data were shown, as in the Canadian study [27], it may not be easy to compare with our data due to the absence of consensus in disease classifications for CLP cases. Another example is that our data pertaining to the reasons for CLP referrals (psychiatric diagnosis) are not available, as they were for a previous study [27, 34], because the DPC database was not designed for specific studies but various research fields. Further efforts for international collaborative research will help improve the quality of available evidence.

Almost 70% of patients who received CLP services used in-hospital psychotherapy; the rests did not use in-hospital psychotherapy (Appendix). This is partly because some CLP services were provided to patients with postoperative delirium which usually disappeared in a short period (i.e., a week), who usually did not need in-hospital psychotherapy. Although the distribution of the number of provided in-hospital psychotherapy sessions was right-skewed, there was another peak in “six and over” during hospitalization. These patients would be those with severe psychiatric conditions or longer lengths of stay. Another possibility is that there was a lack of in-hospital psychiatry in some cases, especially in hospitals where psychiatric healthcare resources are scarce. This is one of the further questions to be addressed.

CLP in Japan started from selected prefectures and gradually spread throughout Japan; however, there are still 14 prefectures where provided no CLP services with their own in 2016 (Fig. 1). In addition, there is a variation in providing CLP services even in the regions (Table 3). Although 5 years had passed from the introduction of CLP, there is still geographical disparity of CLP services, which needs to be improved. It is similar in the United Kingdom, where studies identified widespread availability of liaison psychiatry services in acute care hospitals [35].

The results of the test of independence showed the association between regions and basic variables regarding clinical features (Tables 4 and 5). Although Chi-square test did not provide the strength of the association, the results indicated that the provision of CLP differs among regions. Indeed, there were variations in the proportion of CLP such as for older patients aged 75 and over (11.8 to 30.0%), for planned admission setting (15.6 to 47.1%), and for disease classification (e.g., neoplasm; 4.4 to 30.2%). There results also implied the regional difference in the needs of CLP services, in psychiatric healthcare resources for CLP, and in demand-supply balance in CLP. Investigation in the current/future needs of CLPs and enhancing/expanding the delivery system of CLPs would be considerable.

The results of the multivariate linear regression analysis showed that Tohoku, Shikoku, and Chugoku showed a significant negative correlation with the number of consultations per CLP patient, when compared with Hokkaido, after adjusting for covariates. The results also indicated that several covariates were associated with the number of consultations per CLP patient; for example, i) female patients and younger patients may need more CLP care during hospitalization, ii) the number of consultations per CLP patient may differ among types of diseases, and iii) some patients with longer length of stay may need more CLP care due to severe psychiatric disorders. However, the model explained only 27.4% of the variance in dependent variable, and other factors contribute to the heterogeneity. This implies not only geographic disparity but also unmeasured confounding factors, such as patients’ psychiatric severity, reason for CLP referral, detailed interventions in CLP, timing of CLP, and psychiatric healthcare resources at each hospital. Because CLP is provided to the medical or surgical (somatic) patients, disease category “Mental and behavioral disorders” (most resource-consuming diagnosis) was negatively associated with the number of consultations per CLP patient.

Unfortunately, data regarding CLP in Japan is scarce; even it is not clear how many patients need CLP. It is important to construct database which enables CLP researches. It is not clear whether the impact of CLP differs according to the psychiatric severity or patients’ other clinical background. It is also not clear whether regional psychiatric healthcare resources are associated with the need of CLP or not. The database needs to include patients who need CLP care, regardless of whether they receive CLP, as it could help assess the impact of CLP services on patients’ outcome, such as readmission, patient-reported outcome, cost, and length of stay. Inclusion of variables regarding types of CLP intervention, including patients’ severity, types of psychiatric disorder, and other essential information, is also required for epidemiological investigation. Further efforts for improving geographic disparity are needed for achieving efficient care in CLP services for those who needed care. Further researches for assessing the impact of CLP on outcome, cost-effectiveness, and accessibility are also attractive.

This study has major strengths: it is the largest reported study on this subject in terms of patient numbers in a Japanese setting based on a national administrative database. According to the National Database Open Data, the analysis covered more than 92% of the CLPs in Japan [36]. Further, to our knowledge, this study was the first report which reveals fundamental information of CLP services and geographic disparity in CLP services in Japanese setting which were essential for enhancing the quality of life of patients and improving efficiency in the healthcare delivery system. Thus, our results could inform future interventions to improve medical services and the provision of healthcare.

Several limitations of the present study must be considered. First, this was a retrospective observational study based on an administrative database (DPC). The database covers more than 93% of CLP services conducted across Japan; however, a few hospitals do not participate in the DPC/PDPS system and the exclusion of these hospitals may have introduced an element of sampling bias. The DPC database did not include information whether CLP planning adapted to the needs of patients/hospital/wards are not available. Meanwhile, the CLP team are required to organize treatment plan, explain the plan to the patients, assess the symptoms, and adjusting psychiatric care after discharge. The team also required to write what they do as the team to (electronic) medical record, which are sometimes reviewed by the public agency. In addition, hospitals are required to satisfy facility standards for CLP team (experienced psychiatric staff) to receive fees from providing CLP services. Hence, we speculated that most CLP services adapted to the needs of the patients, however, it is hard to validate this adaption without detailed information which is unavailable in the present study.

Second, data pertaining to several important variables are not available in the DPC database. Therefore, factors such as the difference in psychiatric severity, psychiatric diagnosis before and after CLP, reason for CLP referral, timing of CLP, who provides CLP (doctors or nurses), detailed interventions in CLPs, degree of psychiatrists’ proficiency, and to what extend do the hospitals, psychiatrists and population accept CLP were not included in the analysis. Detailed information regarding psychotherapy was not included neither. We have defined variables based on existing data in the DPC database. Because the DPC database is administrative, we could not add new variables to it for our research purpose. We also could not know number of psychiatrists, preference for CLP in each hospital, and criteria for CLP in each hospital due to the anonymized dataset. In addition, we could not define patients who needs to be received CLP due to the data unavailability. A validation study that defines patients who need CLP is strongly required for assessing factors associated with CLP referral.

Third, the present study did not analyze patient outcomes. Although previous researches had reported the benefit of CLP services [13–17], further outcome studies based on a DPC database would be preferred.

## Conclusions

Data regarding CLP services at the national level had not been analyzed and organized in a way that makes it usable for patients, health care providers, or policy administrators. Our present study revealed the fundamental information and geographical disparity in CLP services in Japan. These results can inform hospital administrators and health service providers improvement the equity of the provision, efficiency of service, and policies relating to healthcare involving CLP. Further research is also needed comparing outcomes of qualifying patients who receive CLP with those who qualify but do not receive these coordinated services and to compare availability, insurance coverage, and utilization of CLP in other countries.

### Supplementary Information

**Additional file 1.** Post hoc analysis of patient charactereistics by region.

## Data Availability

The data availability is not applicable due to an ethical restriction. However, data will be made available by the DPC research group for researchers who meet the criteria for access to these confidential data. Request to access the data should be submitted to the corresponding author.

## Appendix

**Table 7:** Provision of in-hospital psychotherapy in the study cohort

Variables	Fiscal year					Proportion of patients (n) in 2012	Proportion of patients (n) in 2016
	2012	2013	2014	2015	2016		
Psychotherapy (in-hospital)							
0	941	1397	2802	3629	5445	23.6%	31.9%
1	829	1305	1721	2317	3331	20.8%	19.5%
2	551	793	1032	1586	2328	13.8%	13.6%
3	418	497	710	1036	1670	10.5%	9.8%
4	297	343	492	705	1129	7.4%	6.6%
5	209	225	375	509	791	5.2%	4.6%
6+	744	792	1226	1626	2370	18.7%	13.9%

## Abbreviations

CLP: Consultation-liaison psychiatry
DPC: Diagnosis Procedure Combination
DPC/PDPS: Diagnosis Procedure Combination per-diem payment system,
ICD: International Classification of Diseases
IQR: Interquartile Range
OECD: Organization for Economic Cooperation and Development
